# The Importance of the International Community in Protecting the Right to Abortion: The Cases of Malta and of the US Supreme Court

**DOI:** 10.3390/healthcare11040520

**Published:** 2023-02-10

**Authors:** Andrea Cioffi, Camilla Cecannecchia, Luigi Cipolloni, Alessandro Santurro, Fernanda Cioffi

**Affiliations:** 1 Department of Clinical and Experimental Medicine, Section of Legal Medicine, University of Foggia, 71100 Foggia, Italy; 2 Department of Medicine, Surgery and Dentistry, Scuola Medica Salernitana, University of Salerno, 84084 Fisciano, Italy; 3 Department of Anatomical, Histological, Forensic and Orthopaedic Sciences, Sapienza University of Rome, 00185 Roma, Italy; 4 Fertilitas Day Surgery Reproductive Medicine, 84127 Salerno, Italy

**Keywords:** abortion, human rights, bioethics, USA, Europe

## Abstract

According to the World Health Organization (WHO), abortion is often necessary and is not a criminalizable medical act. Unfortunately, despite the global trend in recent years tending towards liberalization of abortion as a fundamental right of women in certain circumstances, it is still not guaranteed in all countries of the world in the same way. Moreover, the abortion debate is often characterized by unscientific opinions based on political and/or religious ideologies. Recently, a European episode has rekindled the debate on abortion: in Malta, a tourist was unable to have an abortion, with consequent risks, even serious ones, on her health. In addition, even in the United States a Supreme Court ruling created a considerable stir: the Roe v. Wade (1973) ruling that had legalized abortion at the federal level was revoked. After the ruling of the Supreme Court, each state of the USA can decide for itself whether and how to legalize abortion. These recent international developments are particularly worrying and make even more evident the need for abortion to be protected at an international level as an inalienable and fundamental human right, and therefore not to be limited.

## 1. Introduction

The World Health Organization (WHO) in the preamble to the Constitution conceptualizes health as ‘a state of complete physical, mental and social well-being and not merely the absence of disease or infirmity’ [1]. In recent years, the WHO has made it clear that respect for the right to health—as a fundamental right—requires that all individuals have access to quality health care, including abortion-related services (health information, assessment and treatment). Preventing and/or delaying timely access to safe abortion is tantamount to harming the psycho-physical and social health status of women. ‘Abortion care is a health care’ is one of the most recent messages launched by Dr Z. Jakab, Deputy Director-General of the WHO [2].

The Third Committee of the United Nations General Assembly—one of the six main commissions of the United Nations that deals with social, humanitarian and cultural issues—in drafting the International Covenant on Economic, Social and Cultural Rights (CESR), did not adopt the same definition of health as contained in the preamble to the Constitution of the WHO. In fact, in article 12.1 of the Covenant—to date ratified by 171 states, and signed but not ratified by Comoros, Cuba, Palau and United States of America [3]—we can read: ‘The States […] recognize the right of everyone to the enjoyment of the highest attainable standard of physical and mental health’ [4]. Although this definition of health is certainly more confined to the concept of the absence of infirmity rather than the overall well-being of the individual, in the Covenant and its subsequent comments there are explicit references to the right to health as an inclusive right not limited to the guarantee of adequate healthcare, but which extends to the guarantee of socio-economic conditions that allow the individual to lead a healthy life (access to education and health information, adequate provision of basic necessities, social and racial equality, combating discrimination, healthy working and environmental conditions, etc.). In 1994, the International Conference on Population and Development (ICPD) Programme of Action (PoA) coordinated by the United Nations reiterated among his principles that: ‘[…] Everyone has the right to the enjoyment of the highest attainable standard of physical and mental health States should take all appropriate measures to ensure, on a basis of equality of men and women, universal access to health-care services, including those related to reproductive health care, which includes family planning and sexual health. Reproductive health-care programmes should provide the widest range of services without any form of coercion. All couples and individuals have the basic right to decide freely and responsibly the number and spacing of their children and to have the information, education and means to do so […]’. Specifically, the ICPD PoA defined the reproductive healthcare as a fundamental right of men and women to be understood as ‘[…] a state of complete physical, mental and social well-being and not merely the absence of disease or infirmity, in all matters relating to the reproductive system and to its functions and processes […]’ [5].

The United Nations has therefore recognized that respect for the right to health cannot be separated from respect for reproductive health care, which States are obliged to protect. This has favored women’s empowerment and marked an important turning point compared to the past, when the reproductive function of women was used in many countries as a tool of political repopulation programs. At the ICPD in Cairo, the States recognized unsafe abortion as a serious public health problem, advocating fair and safe access to all women, in circumstances not contrary to the law [6].

So, as can be seen from the reading of the following General Comment No. 14 of the International Covenant on Economic, Social and Cultural Rights (CESR) [7], to guarantee the provision of maternal health services (ante-partum, intra-partum and post-partum) is to be considered an obligation that cannot be waived: States have the obligation to take concrete and targeted steps towards respect for the right to women’s health in the context of pregnancy and childbirth.

According to international law, in compliance with art. 12 of the CESR, every single State is obliged to guarantee to women—and not only women—the respect of the highest attainable level of reproductive health. As explained in General Comment No. 14 of CESR, the notion of ‘the highest attainable standard of health’ is motivated by the fact that there are unavoidable biological preconditions of the individual (genetic susceptibility to pathologies, choice of adopting unhealthy lifestyles that expose him to the risk of disease) and socio-economic limitations of states that inevitably create unequal standards in the protection of the right to health. It is in fact unrealistic to claim that a State—even the most virtuous from a social-health point of view—guarantees to each individual protection against all forms of ill health, and it is illogical to pretend that States with economically less developed social-health systems maintain the same standards of health care as economically more solid countries. However, admitting a priori a qualitative variability between different states in the protection of the right to health can be risky, especially if not accompanied by periodic checks demonstrating in individual States an effective and operational economic investment in health care, such as to allow the effective achievement of the highest possible standards [8]. Moreover, such achievement is often hampered not only by economic limitations, but also by social, political and religious determinants. The protection of reproductive health lends itself particularly to the risk of very marked discrepancies in the different countries of the world. In fact, it is a field largely contaminated by ideologies that vary not only from country to country, but also within the same country in different historical periods.

The protection of the right to abortion is a fundamental prerequisite for ensuring respect for many of the human rights in the Universal Declaration of Human Rights (UDHR) [9]. As stated at the World Conference on Human Rights held in 1993 in Vienna [10], ‘the human rights of women and of the girl-child are an inalienable, integral and indivisible part of universal human rights’. Moreover, the World Conference on Human Rights has considered inalienable (meaning that no one can take them away) the right of self-determination and has defined the denial of them as ‘a violation of human rights and underlines the importance of the effective realization of this right’. Finally, at the Conference, it was reiterated that all human rights are universal (i.e., inherent to every individual without discrimination), indivisible, interdependent and interrelated; this means that, in order to benefit from a right, it is necessary that all the others are also respected, because all human rights are equally involved in ensuring the protection of human dignity. In fact, as recently reiterated by the WHO—precisely with regard to the protection of the right to health—the improvement of a human right facilitates advancement of others; likewise, deprivation of a right adversely affects others. [11]. The universal nature of human rights implies that, even as importance should be attached to national and regional particularities and to different historical, cultural and religious contexts, it is the duty of States, irrespective of their political, economic and cultural systems, to promote and protect all human rights. [10]. Therefore, art. 21 of the UDHR—which defines the will of the people as the basis of the authority of the government—should be read and interpreted in this light. Although this also constitutes a human right, it should be exercised in compliance with the other rights contained in the UDHR; without the protection of the other human rights, not even that contained in art. 21 can be enjoyed to the full.

Therefore, the states that criminalize sexual and reproductive health services—including abortion—violate the international obligation to respect an inalienable right, which has the same dignity as other rights and which must not be affected by discrimination, political contamination or religious or cultural influences [12,13].

Over the past twenty years, international human rights bodies have provided information on the circumstances in which abortion must be decriminalized. In two landmark judgments, the United Nations Human Rights Committee ruled that Ireland’s strict ban on abortion subjected women to cruel, inhuman and degrading treatment [14,15].

For the first time, in response to an individual complaints procedure, the Committee recognized that criminalizing and prohibiting abortion violates the International Covenant on Civil and Political Rights (CCPR). It is a fundamental treaty in the history of international law and human rights, ratified by 173 states, including the United States of America [16]. The two cases mentioned concerned two women who were denied access to abortion treatment in Ireland despite the discovery of irreversible fetal damage. Because of the ban on abortion in Ireland, women had to go to another country to obtain legal assistance for abortion. In its rulings, the UN Human Rights Committee recognized the psychological, physical and financial harms caused by Ireland’s abortion laws. The Committee found that Ireland had violated Articles 7 (‘No one shall be subjected to torture or to cruel, inhuman or degrading treatment or punishment. In particular, no one shall be subjected without his free consent to medical or scientific experimentation’), 17 (‘No one shall be subjected to arbitrary or unlawful interference with his privacy, family, home or correspondence, nor to unlawful attacks on his honor and reputation’) and 26 (‘All persons are equal before the law and are entitled without any discrimination to the equal protection of the law. In this respect, the law shall prohibit any discrimination and guarantee to all persons equal and effective protection against discrimination on any ground such as race, color, sex, language, religion, political or other opinion, national or social origin, property, birth or other status’) of the ICCPR. According to the Committee, the applicants’ suffering had been further aggravated by the discriminatory conduct of the health services, which did not provide women with clear and detailed information on the possibility of carrying out the termination of pregnancy elsewhere. In this regard, the Commission explicitly adds that ‘women’s reproductive autonomy is included in the right to privacy and may be at stake when the state interferes with a woman’s reproductive decision-making’ [14], as already established in a previous judgment [17]. Moreover, in both cases the suffering of women was aggravated by the need to make a stressful journey and the fact of being forced to live through such a tragic moment in an unfamiliar environment [15].

Therefore, in numerous historical rulings, international judges have stressed that the lack of protection of therapeutic abortion by a country can represent a violation not only of the right to health, but also of the right to privacy and self-determination and, in some situations, the right to be free from cruel, inhuman and degrading treatment. As the Committee points out, the violation of international rights can take place independently of the domestic legal system of individual states.

The numerous interventions on the subject of women’s reproductive health by international human rights organizations have paved the way for widespread abortion liberalization policies. Although in many countries this practice is still limited, in most cases abortion is legally recognized in cases of a woman’s life being at risk, rape, incest or severe fetal impairment [18]. Yet, despite the virtuous example provided by many countries, as well as the repeated recommendations and numerous awareness campaigns of the international scientific community, in 2022 we still live in a context of serious and unacceptable inequity and inequality with regard to accessibility to abortion for women in some countries of the world. Such discrepancies in the protection of the psycho-physical health of women are not exclusively motivated by economic limitations of the health systems of individual states, but also by unjustifiable political and religious interference that makes the respect of a fundamental right questionable, in stark contrast to what is advocated by international jurisprudence.

In 2008 the Parliamentary Assembly of the Council of Europe invited the member state to ‘guarantee women’s effective exercise of their right of access to a safe and legal abortion’ and to ‘lift restrictions which hinder, de jure or de facto, access to safe abortion’ [19], encouraging the spread of abortion liberalization policies in Europe [20]. Abortion is currently legal in many countries in Europe, although there is considerable variability in its restrictions and in the conditions under which this measure is allowed [21,22,23,24]. The most important exceptions are Malta, Vatican City, San Marino, Liechtenstein, Andorra and Poland where abortion is illegal or severely restricted [18,25,26]. This mosaicism predictably forces many women living in countries where laws are more restrictive to resort to life-threatening clandestine abortion [27,28] or to medical tourism in order to secure an abortion [29]; moreover, this heterogenicity translates into serious consequences for the psycho-physical health of women, who, for various reasons (residence, necessity, study and leisure), find themselves having to live in countries where the right to abortion is not respected. For example, during the current Russian–Ukrainian war, many Ukrainian women have been forced to take refuge in Poland, where voluntary abortion is illegal [30,31]. Moreover, recently, an American woman on holiday in Malta—the only European country in which abortion is illegal in any form whatsoever, even for therapeutic reasons, except in cases of imminent danger to the woman’s life—was unable to exercise her right to a therapeutic abortion, with serious repercussions for her state of health [32].

Moreover, in the USA, the unacceptable questionability of respect for the right to abortion only a few days ago made it possible for the US Supreme Court to abolish the historic ‘Roe v. Wade’ ruling, which had recognized abortion as a federal right since 1973 [33]. As of 24 June 2022, every state in the United States can apply its own laws on abortion, effectively entrusting a fundamental human right—comparable to the right to health—to the discretion of the socio-religious positions of political parties or currents of thought [34,35].

In our article, we propose to analyze the serious bioethical and medico-legal implications of the non-respect of the right to abortion in light of most recent threats to its protection. Moreover, we underline the need for an a priori intervention by the European Court of Human Rights concerning those countries which, by prohibiting therapeutic abortion, do not respect women’s right to health. Finally, we consider it a matter of urgency to entrust the international scientific communities with the task of taking super partes decision-making positions on the safety and scientific soundness of therapeutic abortion, a health practice which aims to guarantee respect for the health of women throughout the world regardless of socio-cultural and political differences. The international integration between jurisprudence and scientific evidence is indispensable in order to pursue the achievement of full and universal respect for the inalienable right to reproductive and sexual health of women, according to the directives of international law.

## 2. The Case of Malta, Emblem of European Mosaicism

Malta is the only European country to prohibit abortion for any reason, including in cases of rape, incest, serious abnormalities of the fetus or serious danger to the health of the mother. [21,26]. Maltese law punishes with imprisonment from 18 months to 3 years any woman who voluntarily terminates a pregnancy or anyone who participates in its interruption; health professionals who interrupt the life of the fetus risk up to 4 years in prison, in addition to being disbarred from their professional association [36,37]. Already in 2010, the United Nations’ Convention on the Elimination of All Forms of Discrimination against Women (CEDAW)—a treaty to date ratified by 189 states including Malta and only signed and not ratified by the United States of America and Palau [38]—noted that Maltese women had ‘insufficient access to reproductive health-care services’ [39]. Therefore, in the case LC v Peru, the Committee on the Elimination of Discrimination against Women held that Peru was in breach of its obligations under the CEDAW (i.e., protection to right to health) when it had not allowed a girl of 13 years old to undergo emergency surgery because the procedure required the termination of pregnancy [40,41].

More recently, the United Nations’ Committee on the Rights of the Child (CRC)—a human rights treaty body ratified by all UN member states except the United States of America which is only a signatory [42]—recommends that the State ‘[…] decriminalize abortion in all circumstances and ensure access to safe abortion and post-abortion care services for adolescent girls […]’ [43]. Recently, the Malta Medical Council (MMC) proposed an amendment to the upcoming Equality Bill—an act to prohibit discrimination in various spheres of life—to promote equality also in access to sexual and reproductive healthcare [44,45]. The MMC proposed not to make it mandatory for the health care professional to take part in any medical procedure or treatment that he does not consider in line with his morals if he declares it in advance. In a context such as Malta—where the anti-abortion position among health professionals is particularly entrenched [36]—this amendment would risk creating a dangerous imbalance between the rights of the healthcare professional and patients, constituting an insuperable barrier to access to abortion. It should be remembered that conscientious objection is lawful if it does not adversely affect good medical practice and if it does not cause harm to the patient’s health and cannot in any case be invoked, even if previously declared, in cases of life-saving treatments. In fact, according to the Ethical Guidelines on Conscientious Objection provided by the International Federation of Obstetricians and Gynecologists (FIGO): ‘The primary conscientious duty of obstetrician-gynecologists (hereafter ‘‘practitioners’’) is always to treat, or provide benefit and prevent harm to, the patients for whose care they are responsible. Any conscientious objection to treating a patient is secondary to this primary duty’ [46]. The clause proposed by the MMC would seem to be an attempt to subvert and exploit conscientious objection—a right lawful in certain circumstances—to maintain inaccessibility to abortion, even the therapeutic sort. Despite the recommendations to adapt its laws as soon as possible to protect the inalienable right to women’s reproductive health, restrictive policies on abortion are deeply rooted in Malta, continuing to favor tragic everyday scenarios even in recent cases, as we describe.

A 38-year-old, 16-weeks pregnant American woman, while on babymoon in Malta with her husband, began bleeding. A gynaecological examination showed severe placental abruption and preterm rupture of the amniochorionic membranes (pPROM), which are indications of incipient miscarriage; the physicians informed the couple that the pregnancy would not continue for long, and that the fetus would not survive. Medical management of pPROM must be the result of balancing the potential benefits to the new-born from continuing the pregnancy against the risk of infection and serious health consequences for the mother, who is at high risk of infection. Indeed, in such circumstances, the foetal mortality rate is very high. This risk, in the specific case, was even higher since the fetus was only 16 weeks old and far from acquiring even a potential capacity for survival outside the maternal uterus [47]. Conversely, the infectious (hence also life-threatening) risk to the mother was particularly high, necessitating an immediate emptying of the uterus to avoid serious short- and/or long-term complications. However, the Maltese law prevents doctors from intervening and/or participating in the voluntary termination of pregnancy, even in cases of serious danger to the health of the mother, as in this case. Therefore, since the foetal heartbeat was still detectable, the doctors did not carry out the abortion, even though this was the only and most effective therapeutic tool to avoid serious repercussions concerning the health of the woman, who was at high risk of death from sepsis. Fortunately, after more than a week, the couple obtained the consent of the local authorities to leave the country due to a situation defined by the Maltese authorities as ‘life-threatening’. The transfer to Spain—only partially reimbursed by the couple’s health insurance—was hindered by the difficulty of finding an airline that would take on the criminal consequences of the case [48]. To date, the woman’s life is not in danger, but this does not exclude the possibility that the delayed medical intervention may have produced irreversible results on her psycho-physical health. The Maltese episode brings to light the tragic case, in 2012, of an Irish woman of Indian origin, pregnant at 17 weeks, who died of septicaemia after the Irish doctors refused to terminate a miscarriage due to persistent foetal heartbeat, as required by law in Ireland [49]. These tragic cases confirm that abortion is often necessary to preserve a woman’s life and psycho-physical health. In the event that the continuation of pregnancy constitutes a serious risk to the health of the mother, health care providers are obliged to interrupt it with respect for her right to health, while making every effort to try to keep the fetus alive. This is all the more so the case where, as in the cases described, the mother’s psycho-physical health is at risk, and the fetus would have no independent life outside the womb. In such circumstances, there is no scientific reason not to resort to therapeutic abortion since there is no autonomous life to be preserved except that of the mother. It should also be specified that abortion is to be considered licit even when there is no immediate risk to the mother’s life, but the continuation of the pregnancy constitutes a serious danger to her state of health (physical or psychological): even in such circumstances, it cannot be forgotten that the mother is the only human being legally alive and, as such, the sole depositary of an inalienable right—that of health—which must be guaranteed [50,51]. This is also considering that pregnancy-related diseases often have an unpredictable course, and their outcome is greatly influenced by the timeliness of therapeutic intervention. The non-accessibility or delayed accessibility to therapeutic abortion—if not scientifically justified—can produce irreversible medical-legal consequences with respect to the psycho-physical health of the woman. In the case of the recent Maltese episode, the lack of timely therapeutic intervention may have resulted in gynaecological damage that would hinder a possible future pregnancy, which is already undermined by the woman’s advanced age and previous miscarriage [52]. Maltese legislative and bureaucratic limitations prevented the woman from having a safe therapeutic abortion, imposing the use of an untimely surgical intervention, which was associated with a greater risk of complications and lower chances of survival because it was implemented in conditions of greater clinical instability. Moreover, the woman was forced to undergo prolonged hospitalization and treatments, which would have otherwise not been required if the therapeutic procedure had been performed as scientifically indicated. Moreover, one cannot overlook the psychological outcomes that this tragic event will produce not only on the mother but also on her partner. Socio-religious and scientifically unfounded interferences forced the couple to live, for days, with the hope that their son’s heart would stop, this being the only condition of survival for the woman. This stressful circumstance—preventable and avoidable—was, in addition to that linked to the premature termination of pregnancy, already in itself associated with serious risk of repercussions– not always preventable—on the parents’ psychological health [53,54].

The Maltese episode must be the last such event to demonstrate the need to homogenize European legislation on abortion, on the basis of clear guidelines provided by international law. In addition, we consider it necessary for the European Court of Human Rights to intervene a priori with severe sanctions against those states which do not comply with the Convention for the Protection of Human Rights and Fundamental Freedoms, better known as the European Convention on Human Rights (ECHR), ratified by all 46 Member States of the Council of Europe [55]. In fact, the European Court of Human Rights—an institution of the Council of Europe—has jurisdiction to rule on individual or state applications alleging violations of the rights set out in the European Convention on Human Rights. The right to therapeutic abortion is, for all intents and purposes, part of the many principles in the Treaty, first of all the ‘Obligation to respect Human Rights’ (Art.1), ‘Right to life’ (Art. 2) and ‘Right to liberty and security’ (Art. 5). Moreover, the right to therapeutic abortion also falls within the ‘Right to respect for private and family life’, for which art. 8 specifies that ‘There shall be no interference by a public authority with the exercise of this right except such as is in accordance with the law and is necessary in a democratic society in the interests of national security, public safety or the economic well-being of the country, for the prevention of disorder or crime, for the protection of health or morals, or for the protection of the rights and freedoms of others’ [56]. Therefore, European treaty bodies seem to ensure protection of the rights of pregnant women, despite not officially recognizing the right to abortion. Unfortunately, the international legal bodies called upon to rule on the violation of human rights have not always shown a position of guarantee. In particular, the analysis of the judgments of the European Court of Human Rights on abortion reveals that the Court has reached very different conclusions depending on the action. In fact, on the one hand, in many cases where the ban or the delay of the voluntary interruption of pregnancy has produced serious consequences for the health of the woman and/or the newborn, the judges of Strasbourg have recognized a violation of art. 8 of the ECHR by the State [57,58,59].

On the other hand, when the Court was called upon to rule on the possibility that the prohibition of abortion, in certain circumstances, constitutes a violation of the content of the ECHR, it held that the action was unfounded. We refer to the historic judgment A., B. and C. c. Ireland of 2010, which is notably useful in examining the Court’s position on this issue [60]. Three young women have appealed to the European Court of Human Rights because the anti-abortion legislation in force in Ireland had forced them to travel to Great Britain to terminate their pregnancy, with serious physical and psychological damage. In fact, the necessary travel, carried out in precarious conditions and with scarce economic resources, delayed access to abortion and caused real damage to their health. For this reason, the three applicants claimed infringement of art. 3 (inhuman and degrading treatment) and art. 8 (right to respect for private and family life) of the ECHR; moreover, the third woman also claimed the violation of art. 2 (right to life), since she was already seriously ill with a rare form of cancer (continuation of pregnancy would not have allowed her to continue life-saving chemotherapy). In all three cases, the international courts have rejected the appeal on the violation of art. 3 because the damage suffered by the women was not ‘serious enough’ to be equated with inhuman and degrading treatment. Regarding the alleged violation of art. 8 and art. 2, the Court ruled in favor only of the third applicant suffering from cancer, because she was the only one at real risk. The Court justified the rejection of the first two appeals by the absence of a political and scientific consensus among Member States on the beginning of life and thus of a single position on what should prevail, whether the unborn child’s right to life or the woman’s right to abortion. In other words, the Court stressed that its role does not involve taking a position on the legitimacy of abortion: each State, based on its historical and cultural heritage (therefore also not on the basis of scientific evidence), is free to recognize or not the right to abortion as prevailing over the right of the unborn child.

The wide decision-making discretion granted by the Court to individual States in the matter of abortion also had a considerable impact on the decisions taken by the Court, especially on the appealed inhuman and degrading treatment. One cannot help but notice that international judges recognize a violation of art. 3 only in those cases where access to abortion, while being guaranteed by domestic law, is rendered impracticable by factual circumstances. In contrast, when abortion is not allowed under national law, the Court appears reluctant to recognize such a violation. This leads inevitably to the question: is a possible appeal to the European Court useful for the recognition of a universal right, regardless of the country of origin? In the event of an appeal to the European Court, how would the case of Malta be judged? In this case too, the Maltese State applied its national law which provides for a ban on abortion in all circumstances. So, what is the limit (in terms of risk to the psycho-physical health of women) beyond which state laws become surmountable? The excessive discretion entrusted to the States in the matter of abortion—which also affects the decisions of international judges—risks undermining its role as guarantor of human rights. This risk is due to the fact that the internal policies of individual states in the field of abortion are not only based on scientific, but also on cultural, moral and religious bases. To minimize this risk, we consider it extremely urgent for the international community to formulate and impose on all states a shared operational protocol based on constantly updated scientific evidence that protects women’s fair and safe access to therapeutic abortion. To intervene a priori with integration between international law and science would allow reaching, in the various countries, the highest standards in the protection of sexual and reproductive health. We believe, in fact, that the imposition of scientific protocols to protect the right to health of pregnant women—together with a rational and lawful accessibility of health professionals to conscientious objection—is a valid tool to prevent the respect of inalienable human rights (recognized in international law) from being contaminated by political, cultural and religious ideologies. As discussed above, in cases where it is necessary to resort to abortion due to risk to a woman’s life, the invocation of conscientious objection by healthcare professionals should be made illegal at the international level (especially in the absence of a colleague of equal competence who can replace himself promptly). Moreover—in order to respect the right to health—it is necessary that even States in which abortion is prohibited or severely restricted guarantee health care to women in the period before and after an abortion (even if the procedure is performed in another country).

We consider essential the formulation of a declaration outlining the scientific consensus among states concerning therapeutic abortion; the latter is a universal right of women and of the whole community and cannot be delegated to the domestic order of the individual State without a scientific basis. Only after securing this can each individual State’s legal system express its own orientation on the limitations of abortion. This consensus is also essential to promote the urgent harmonization of therapeutic abortion among all countries, consistent with the universality of human rights. Finally, in our opinion, this consensus is important to promote the urgent homogenization on therapeutic abortion among all countries, consistent with the universality of human rights.

Before this happens, it is urgent to at least offer protection to pregnant women who travel to countries where therapeutic abortion is illegal or severely restricted. To this end, first, we consider it necessary for pregnant women to be adequately informed before they leave (possibly in writing on a form) of the limitations in terms of health care which they will face if they stay in countries where therapeutic abortion is illegal. If it is not possible to guarantee the presence of international health facilities that will perform therapeutic abortions independently of local legislation in these countries, pregnant women should be clearly advised against choosing such destinations. Ensuring this information for pregnant women and their partners is crucial to coping with this transition phase, which risks producing irreversible health damage. Therefore, signing an information notice could make the woman and partner more knowledgeable in choosing a travel destination during the gestation period. To the same end, it would be necessary to encourage greater awareness of the issue in the community; in fact, especially at this phase, it is important for women to be more aware of the serious health consequences that the lack of lack of homogeneity in the legalization of abortion in various countries can potentially bring with it. In addition, especially where the reason for travel is one of necessity (work, emergency), it is necessary that the woman’s travel health insurance fully covers, where necessary, the costs of travel to countries where therapeutic abortion is legal. This is essential to prevent the right to therapeutic abortion—and hence the human right to psychological and physical health—from becoming an elitist right, exclusive to economically better-off women [61].

## 3. US Supreme Court: Abortion, from Inalienable Right to Political Option

In 2018, the Jackson Women’s Health Organization, an OB/GYN clinic in Mississippi, challenged the constitutionality of the ‘Gestational Age Act’ [62] in federal court. The law, enacted on 19 March 2018, prohibited abortions after the 15th week of gestation ‘except in cases of medical emergencies or in the case of severe fetal abnormalities’. On 24 June 2022, the US Supreme Court ruled on the case by overturning Roe v. Wade [33], which had guaranteed the federal right to abortion since 1973. In Dobbs v. Jackson Women’s Health Organization, the judges of the Court vindicated the absence of the right to abortion in the US Constitution, restoring the authority to legalize, prohibit and regulate abortion to the elected political representatives of the individual states [26]. The Court—among the questionable arguments of the judgement—underscores that women ‘are not without electoral or political power’ and that they will still be able to express their preference on the ‘abortion issue’ through their vote. In this sense, it is good to remember that abortion is not a ‘women’s’ right but is, for all intents and purposes, part of the human right to psycho-physical health, which is independent of gender and in respect of which the balance and well-being of the entire community is based. Is it therefore reasonable to entrust to political majorities the possibility of prohibiting and/or establishing limits on the accessibility of abortion? Can a human right be the subject of political debate? History is teaching us that this is tragically possible today. We believe that there is an urgent need to update international law regarding human rights, the principles of which must be respected by all states in the world regardless of socio-cultural and political orientation. Furthermore, in the current tragic scenario in the United States, it is necessary to establish limits to the decision-making power of individual states on the issue of abortion. It must be possible for these limits to be established and constantly updated by the international scientific communities—the only ones competent to scientifically establish under what circumstances the interruption of pregnancy is a necessary condition for preserving a woman’s psycho-physical health. Who and on what basis establishes the gestational week beyond which voluntary abortion is illegal? What is a medical emergency in which therapeutic abortion would be permitted? What is the probability of death of the mother beyond which a legal decision to intervene by terminating the pregnancy can be taken? It is essential to entrust the scientific community with the task of establishing, at the international level, all the conditions in which therapeutic abortion constitutes a medical and/or surgical indication. If the intention is to allow individual states to regulate the law on abortion based on socio-cultural and political inclinations, there is no way we can fail to guarantee respect for the psycho-physical health of the human being, which is independent of any conditioning whatsoever. It will therefore be necessary to entrust scientific communities with the task of defining recommendations on the use of abortion in various circumstances. For example, recourse to the termination of pregnancy may be necessary not only in conditions of imminent risk to the woman’s life; during gestation, the mother may experience decompensation of a chronic pathology or diagnosis of a new pathology which—albeit not constituting a health emergency—requires fetotoxic pharmacological treatments (antineoplastic drugs, methotrexate, valproic acid) or surgical treatments incompatible with the continuation of gestation but necessary to increase the mother’s hope of long-term survival. Do such serious conditions of risk to the health of the woman fall within the so-called ‘health emergencies’? The same applies to the risk of ‘severe foetal abnormalities’. What is meant by this? What is the limit beyond which a foetal abnormality can be considered such as to permit therapeutic abortion after the 15th week of gestation? These clarifications are urgently needed to prevent unresolved questions from resulting in irrational and anti-scientific management of the right to health.

What the US Court ruled is considered unscientific and above all in contrast with the only certainty inherent in the issue of abortion: abortion is not a political or legislative choice but an inalienable right.

## 4. Conclusions

On 8 March 2022, the WHO published new guidelines on abortion [63] based on the latest scientific evidence and aimed at ensuring access to safe abortion for women and girls worldwide, helping to prevent the over 25 million unsafe abortions that occur each year. Among the main recommendations provided by the WHO is that of decriminalizing abortion, favoring pharmacological abortion over surgical abortion, making it as accessible as possible and speeding up and facilitating its use (reducing compulsory waiting times from the first request and correcting delays and/or limitations linked to conscientious objectors). For the WHO, abortion is to be considered, for all intents and purposes, an often-necessary medical act, fair access to which is essential to avoid serious damage to the psycho-physical health of women. Banning abortion does not reduce its incidence; on the contrary, it opens the way to clandestine abortions that do not meet safety and hygiene standards and constitute a risk to public health [64] as well as a very high risk of morbidity and mortality for women [65]. On the other hand, limiting and/or prohibiting access to abortion leads to an increase in the continuation of unwanted pregnancies—especially in those countries with less access to safe means of contraception for cultural and economic reasons. Moreover, the risk of unwanted pregnancies and their related consequences are particularly high among poorer women who cannot afford the cost of moving from a country where abortion is illegal to one where it is. Denying access to abortion inevitably reflects on both the psychological and physical health of the unwanted child and the mother [66]; scientific evidence shows that unplanned/unwanted pregnancy is among the predictors of postpartum depression as well as of factors that may influence the decision to abort (marital and socioeconomic status) [67]. Moreover, denying abortion seems to have consequences not only on the psycho-physical health of the unwanted baby [68,69] but also on the psycho-physical development of the siblings [70].

Therefore, what has been argued demonstrates that the right to abortion is a human right for all intents and purposes, and it concerns the woman only in the first instance; in fact, failure to respect it has serious consequences for the entire community at various levels. To guarantee therapeutic abortion, it is necessary for abortion to be enshrined as a fundamental human right. This would be a fundamental step to ensure that the right to therapeutic abortion become universal and equally guaranteed in all countries. Furthermore, official recognition of therapeutic abortion as a ‘human right’ would make it possible to officially recognize that the right to therapeutic abortion is aimed at protecting not only women’s health, but also that of the partner, of the children and, therefore, of the whole community (as scientifically proven). Our study has shown a lack of uniformity in international law on abortion, certainly motivated by the fragmentation of the numerous treaties, subject to varying interpretations, and not always ratified by all States. We believe that a dialogue between the scientific community and international law is a fundamental tool to homogenize the right to therapeutic abortion and to prevent it from being subject to political, cultural and religious influences. In our opinion, greater effort by the international scientific community and supranational bodies will be essential and should be aimed at establishing a ban on restricting access to abortion. Indeed, the concrete involvement of the scientific community could be a key to ensuring that international law can also be respected when it comes to abortion. The presence of international scientific protocols would not only make it possible to guide legal bodies more homogeneously in the event of appeals, but also to legitimize more stringent measures against non-compliant States. In this regard, similar to what is done with economic sanctions against countries that do not respect international law in the military sphere, we advocate for stronger interventions and sanctions against those countries that do not respect human rights related to abortion. The international community cannot use double standards when it comes to human rights which, as argued, are to be considered universal, indivisible, interdependent and interrelated.

It is well known that the right to abortion raises numerous bioethical debates, especially regarding respect for life as such and the personality of the embryo [71,72,73,74]. We certainly do not want to underestimate the importance of the bioethical debate on abortion. In fact, restrictions on access to abortion are not only a scientific issue, but also a moral one, especially regarding the rights of the fetus and the unborn. However, we believe that, especially when it concerns universal human rights, the bioethical debate should be adequately supported by scientific evidence. Indeed, in the case of a scientifically proven psycho-physical risk to the mother linked to the continuation of pregnancy, it should be agreed upon that the right to women’s health must be respected. We believe that, regardless of bioethical positions, we all agree to preserve women’s health if it is seriously at risk. This is especially true if the gestational age, as in the cases described, is not scientifically compatible with life. Although we consider the bioethical debate on the subject to be desirable and useful, to date we have not reached an unambiguous bioethical position about the beginning of life. As stated above, the European Court of Human Rights justified the rejection of two appeals by the absence of a political and scientific consensus among States on the beginning of life and on embryos’ rights. In our opinion, however, this cannot legitimize the choice of entrusting the management of limitations to abortion (strictly related to respect for human rights) to the political and ideological inclination within each state. In other words, the current lack of a bioethical and scientific consensus on the beginning of life cannot be a valid reason to leave room for political, social, cultural and religious contaminations that are unacceptable when it comes to human rights. Moreover, this cannot risk becoming a reason to make debatable scientific evidence about the risk to the psycho-physical health of the woman in cases of a ban on abortion in certain circumstances. To date, we are still far from agreeing on the bioethical issues mentioned, but this fragmentation is not acceptable in cases where abortion is scientifically necessary to protect the right to psycho-physical health of women. Therefore, the scientific community should participate not only in the aim of reaching a definitive position on the beginning of life, but should also unambiguously establish the circumstances in which the continuation of pregnancy entails serious consequences on psycho-physical health of women. In our opinion, international scientific bodies play a key role in determining the circumstances in which abortion should be practiced for the protection of women’s health, obviously always respecting the right balance with the health status of the fetus, from a scientific point of view. Pending a bioethical and scientific consensus about the beginning of life, it is especially important that evidence already scientifically validated does not risk being contaminated by anti-scientific interference. We therefore consider it urgent for the scientific community and international law to take a stronger stance on this issue.

To achieve a concrete objective, it is essential to understand that abortion is not a political, social, economic, religious or cultural choice but an inalienable and fundamental human right. Limiting or prohibiting access to abortion is the same as limiting or prohibiting access to basic medical care. Political–religious–social interference in the delineation of the practice of abortion cannot be tolerated. We therefore believe that the findings of the scientific community concerning reproductive health should be integrated with international law and therefore imposed on all states, in order to guarantee all women a fair protection of the right to reproductive and sexual health. Moreover, imposing a protocol that scientifically legitimizes women’s access to abortion in established circumstances is a fundamental tool to counter entrenched anti-abortion movements. They have a serious and dangerous impact on women, who, by virtue of totally anti-scientific ideologies, sometimes end up fighting themselves against their inalienable rights. Only the scientific community can be the glimmer of light in the research and identification of possible limits and specific modalities of abortion (medical and surgical; therapeutic and voluntary) in the absence of a ‘mind-numbing’ ideological conditioning.

## Data Availability

The data analyzed during this study are included in this published article. Further inquiries can be directed to the corresponding author.
